# Fast and Simple Microwave Synthesis of TiO_2_/Au Nanoparticles for Gas-Phase Photocatalytic Hydrogen Generation

**DOI:** 10.3389/fchem.2018.00110

**Published:** 2018-04-12

**Authors:** Anna May-Masnou, Lluís Soler, Miquel Torras, Pol Salles, Jordi Llorca, Anna Roig

**Affiliations:** ^1^Institut de Ciència de Materials de Barcelona, CSIC, Bellaterra, Spain; ^2^Departament d'Enginyeria Química and Barcelona Research, Center for Multiscale Science and Engineering, Institut de Tècniques Energètiques, Universitat Politècnica de Catalunya, EEBE, Barcelona, Spain

**Keywords:** nanotitania, gold nanoparticles, microwave synthesis, gas phase photocatalysis, hydrogen production

## Abstract

The fabrication of small anatase titanium dioxide (TiO_2_) nanoparticles (NPs) attached to larger anisotropic gold (Au) morphologies by a very fast and simple two-step microwave-assisted synthesis is presented. The TiO_2_/Au NPs are synthesized using polyvinylpyrrolidone (PVP) as reducing, capping and stabilizing agent through a polyol approach. To optimize the contact between the titania and the gold and facilitate electron transfer, the PVP is removed by calcination at mild temperatures. The nanocatalysts activity is then evaluated in the photocatalytic production of hydrogen from water/ethanol mixtures in gas-phase at ambient temperature. A maximum value of 5.3 mmol·${\text{g}}_{\text{cat}}^{-1}\cdot$h^−1^ (7.4 mmol·${\text{g}}_{\text{TiO}2}^{-1}\cdot$h^−1^) of hydrogen is recorded for the system with larger gold particles at an optimum calcination temperature of 450°C. Herein we demonstrate that TiO_2_-based photocatalysts with high Au loading and large Au particle size (≈50 nm) NPs have photocatalytic activity.

## Introduction

The prospect of achieving clean and renewable hydrogen at ambient temperatures through the photocatalytic water splitting reaction has gained much attention since the pioneer work of Honda and Fujishima in 1972 using TiO_2_ as photocatalyst in a photoelectrochemical cell (Fujishima and Honda, 1972). TiO_2_ is an excellent candidate for photocatalysis presenting an effective generation of electron-hole pairs, chemical stability to corrosion and photocorrosion and providing suitable interfaces for charge transfer. However, its large band gap (anatase 3.2 eV; rutile 3.0 eV) restricts its use in the UV fraction of the spectrum, and the fast recombination rate of the charge carriers (electron-hole) after excitation compromises the efficiency of H_2_ generation (Ge et al., 2017).

Higher photocatalytic activities have been achieved using more complex nanocatalysts, for instance, by doping TiO_2_ with cations or anions or by combining the TiO_2_ with metal or semiconductor nanoparticles (NPs). Usually noble metal NPs, such as Au (Bamwenda et al., 1995; Primo et al., 2011; Jovic et al., 2013a,b; Taboada et al., 2014a,b), Pd (Khojasteh et al., 2016), Pt (Jovic et al., 2013b; Al-Azri et al., 2015), Au-Cu (Bonmatí et al., 2015), Au-Pd (Su et al., 2014), or metal oxide NPs, like RuO_2_ or CuO (Bandara et al., 2005; Xu and Sun, 2009; Yu et al., 2011) have been used. These doping agents act as a cocatalyst, enhancing the electron-hole charge separation, avoiding or delaying the recombination rate and increasing the overall photocatalytic activity. However, this technology is still not fully commercially available, mainly due to the low efficiency of the materials, to their low stability during the water splitting reaction, and to its overall high cost.

The influence of metal loading has been studied thoroughly considering small metal percentages, usually up to 10 wt%. In this range, the hydrogen yield increases with increasing metal content, reaches a maximum, and then starts a steady decrease. The optimum loading is usually found between 0.5 and 8 wt% (Murdoch et al., 2011; Jovic et al., 2013a). A common reasoning is that the surface of the semiconductor becomes partially blocked by the metal, decreasing the surface concentration of electrons and holes available for reaction, and hindering light absorption (Bamwenda et al., 1995; Maeda, 2011). Another explanation of the photocatalytic activity decrease at large metal fractions is that the deposited metal particles can themselves act as recombination centers for the photogenerated electrons and holes (Bamwenda et al., 1995). The influence of particle size has also been widely studied. Most authors sustain that the catalytic activity of Au NPs disappears for particles larger than 20 nm, since the activity is correlated with the number of Au atoms on the external surface (Haruta, 1997; Primo et al., 2011). Accordingly, Murdoch et al., 2011 showed that Au NPs in the size range 3–30 nm (especially up to 12 nm) on TiO_2_ were the most active in hydrogen production. However, the optimum metal particle size will also depend on the size of TiO_2_ NPs and on the type of contact with the semiconductor support. Thus, full implications of the loading, size, and shape of the metal cocatalyst need to be further explored.

The overall activity of the photocatalyst and hydrogen production can also be enhanced by the addition of easily oxidizable sacrificial agents, acting as electron donors (Nadeem et al., 2010). According to previous works (Taboada et al., 2014a,b; Bonmatí et al., 2015), some of us demonstrated that the rate of H_2_ production increased due to the irreversible oxidation of the organic molecule with the holes and the concomitant suppression of electron-hole recombination. Although ethanol is not the sacrificial agent with the highest rate of hydrogen production (Bowker, 2012; Taboada et al., 2014a; Chen et al., 2015), it is by far one of the most promising and used, since it is ready available, easy to transport, safe to handle, and it can be produced by renewable biomass. Ethanol is a good option when performing gas-phase reactions, since it can be easily mixed with water and form gaseous mixtures. Moreover, in gas-phase reaction, contrarily to a liquid-phase reaction, as the H_2_ and O_2_ formed on the surface of the catalyst are rapidly released, there is shorter time for the formation of undesired byproducts. However, acetaldehyde is produced as a result of ethanol dehydrogenation, as stated in Equation (1) (Taboada et al., 2014b).

(1) $$\begin{array}{r} \text{C}{\text{H}}_{3}\text{C}{\text{H}}_{2}\text{OH}\to \text{C}{\text{H}}_{3}\text{CHO}+{\text{H}}_{2} \end{array}$$

Producing functional materials with better properties than the existing ones, while using cheaper, faster and cleaner synthesis, is in high demand. The most widespread methods to obtain nanoparticles, such as co-precipitation, thermal decomposition and microemulsion are limited by either the amount of available reagents or the required long unpractical processing times. Microwave energy is becoming an increasingly attractive alternative tool in all areas of synthetic chemistry because it can boost some competitive advantages over other fabrication methods. It is fast, produces high yields, is scalable and is easy to operate, being efficient in terms of energy consumption and environmentally friendly (Michael et al., 1991; Stuerga et al., 1993; Bilecka and Niederberger, 2010). In particular, the versatility of the method for the synthesis of nanoparticles has been reported (Stuerga et al., 1993; Baghbanzadeh et al., 2011). Monodisperse nanoparticles are achieved due to more homogeneous inner core heating with no solvent convective currents due to temperature gradients (Baghbanzadeh et al., 2011), which decreases the possibility of asynchronic nucleation and heterogeneous nanocrystal growth. Indeed, microwave-assisted synthesis has appeared as an attractive way to prepare scalable, uniform and controllable colloids with complex kinetic/thermodynamic control over crystallization processes (Hachtel et al., 2016).

Herein, we report on a facile synthesis of TiO_2_/Au nanostructures through a microwave-assisted route (Gonzalez-Moragas et al., 2015; Yu et al., 2015; Hachtel et al., 2016). The obtained nanomaterials consist of two different sizes of Au NPs (*ca*. 50 and *ca*. 10 nm) in contact with smaller crystalline TiO_2_-anatase NPs of *ca*. 10 nm in both cases. We then investigate and compare the photocatalytic performance of the TiO_2_/Au nanomaterials with Au loading as high as 20 wt%. The photocatalytic activity is studied on H_2_ production from a mixture of ethanol and water in gas phase. We analyzed the effect of the calcination temperature on the photocatalytic efficiency of the two TiO_2_/Au nanostructures. The use of the same size of TiO_2_ NPs and varying the size of Au NPs allows us to elucidate the role of the size of Au NPs/contact points. There are very few previous studies analyzing the photocatalytic activity of TiO_2_ NPs with very high Au loading and large Au particle size, which by using smaller TiO_2_ NPs could increase the number of Schottky junctions and, therefore, improve the overall photocatalytic performance of the process.

## Materials and methods

### Materials

Titanium butoxide (TBOT) (97%), polyvinylpyrrolidone (PVP, average molecular weight: 10000 g/mol) and hydrogen tetrachloroaurate trihydrate (HAuCl_4_·3H_2_O ≥ 99.9%) were purchased from Sigma-Aldrich. Anhydrous benzyl alcohol (99%) and pure ethanol (> 99.9%) was purchased from Scharlau, and ethylene glycol (EG ≥ 99%), HCl (37%) and acetone were purchased at Panreac. All materials were used as-received without further purification. Milli-Q water (MQ-H_2_O) was used in all experiments.

### Synthesis of PVP coated TiO_2_ NPs

TiO_2_ NPs with a PVP surface coating are synthesized adapting our microwave (MW) synthesis protocol for superparamagnetic iron oxide NPs (SPIONs) (Pascu et al., 2012; Yu et al., 2015; Hachtel et al., 2016) in a CEM Discover reactor (Explorer 12-Hybrid) at a frequency of 2.45 GHz and 300 W of power. Moreover, the synthesis process is scaled-up a factor of 4 in order to have enough quantity of material. Briefly, 2.72 g PVP (0.272 mmol) are dissolved in 16 mL of anhydrous benzyl alcohol (BA) by continuous sonication. Then, 240 μL TBOT (0.684 mmol) are mixed with the above prepared solution to give a homogeneous solution of a yellowish color. The tubes are then placed in the MW reactor and heated first to 50°C for 5 min to ensure a complete solubilization of the precursor, and then at 190°C for 10 min. The final solution is dark-yellow and no precipitate is observed, indicating that the NPs are dispersed in the solution. TiO_2_ NP are collected by adding 35 mL acetone in 4 mL of the solution (4 tubes) to precipitate the NPS and remove the excess PVP, centrifuging at 6,000 rpm for 30 min twice, and redispersing the precipitate of each tube in 16 mL ethylene glycol (EG) to be further used.

### Synthesis of TiO_2_/Au NPs

**Figure 1:**
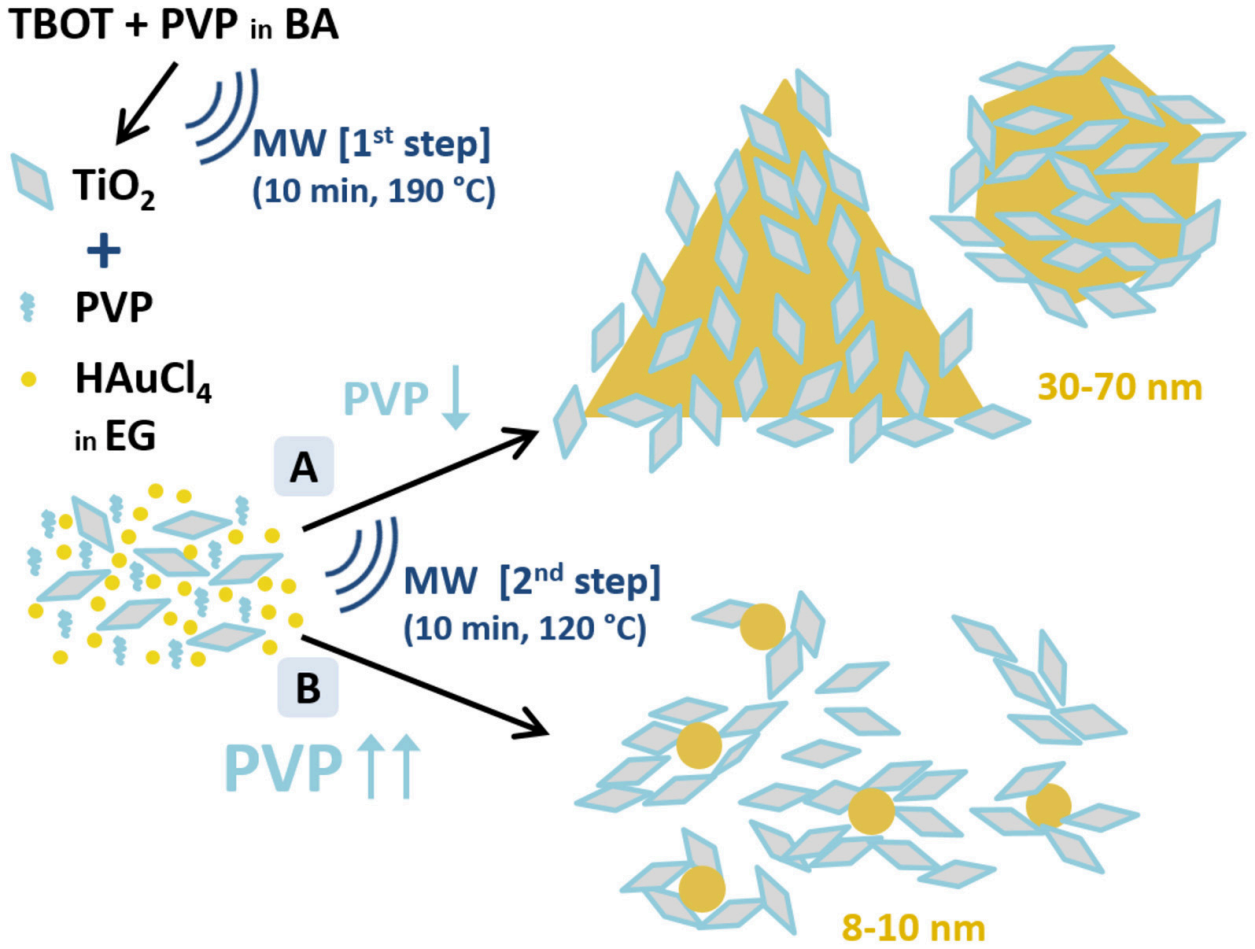
Schematic representation of the two-step microwave-assisted synthesis of Au/TiO_2_ nanostructures. A and B depict the two performed synthetic routes to obtain Au and TiO_2_ nanoparticles with different Au particle size distributions. (A) TiO_2_:PVP:HAuCl_4_ = 1:0.097:0.155; (B) TiO_2_:PVP:HAuCl_4_ = 1:0.583:0.155 (molar ratios). Notation: TBOT, titanium butoxide; PVP, polyvinylpyrrolidone; BA, benzylalcohol; HAuCl_4_, hydrogen tetrachloroaurate; EG, ethylene glicol; MW, microwave-assisted synthesis.

Two different synthetic routes were investigated (A and B, see Figure 1). The only variation between the two syntheses is the quantity of PVP added: 100 mg of PVP in synthesis A and 600 mg in synthesis B. PVP is added to the as-obtained TiO_2_ dispersions in EG (16 mL) and sonicated to obtain a homogenous mixture, followed by the addition of 64 μL of 250 mM HAuCl_4_·3H_2_O (0.016 mmol). The molar ratio of free PVP to HAuCl_4_ is 0.625:1 in synthesis A and 3.75:1 in synthesis B; and of TiO_2_ to HAuCl_4_ is 6.41:1 in both syntheses. The solution is heated at 120°C for 10 min in the microwave reactor. The final solution is tyrian-purple and no precipitate is observed. As-obtained TiO_2_/Au are washed twice with acetone (35 mL acetone in 8 mL of solution, 2 tubes) to remove free PVP, and collected by centrifugation at 6,000 rpm for 30 min twice. The samples are redispersed in 2 mL of MQ-H_2_O and kept for further characterization. In total, we will have 4 mL of sample A and 4 mL of sample B. Part of the sample is dried overnight at 100°C, and other parts are annealed in air at 400, 450, 550, or 600°C for 2 h at a heating rate of 2°/min to remove the organic PVP and increase the contact between Au and TiO_2_.

### Purification of TiO_2_/Au NPs

To remove the excess TiO_2_ not bonded to the Au NPs, the as-synthesized particles dispersed in 2 mL MQ-H_2_O are divided into two Eppendorf tubes with 1 mL each and centrifuged at 6,000 rpm during 25 min (synthesis A) and 4 min (synthesis B). The supernatant is discarded, another 1 mL MQ-H_2_O is added, and the centrifugation repeated. This procedure is repeated two more times for synthesis A during 15 min each. The supernatant is further discarded and the total resulting solid is redispersed in 2 mL MQ-H_2_O.

### Materials characterization

Ultraviolet-visible-near infrared (UV-Vis-NIR) spectra were collected on a Varian Cary-5000 UV-Vis-NIR spectrophotometer between 350 and 800 nm. The morphologies and crystalline phase of the Au and TiO_2_ NPs were analyzed in a JEOL JEM-1210 transmission electron microscopy (TEM) operating at 120 KV. One drop of the NPs dispersion was placed in a carbon film copper grid. High resolution TEM (HRTEM), high angular annular dark field scanning transmission electron microscopy (HAADF-STEM) images, and STEM-Energy dispersive X-ray spectroscopy (STEM-EDX) profiles were acquired using a FEI Tecnai G2 F20 microscope operated at 200 kV equipped with an EDAX super ultra-thin window (SUTW) X-ray detector, on the as-synthesized and calcined at 450°C samples. Elemental analysis of C, H, N and S was performed through combustion of the samples at 1,200°C in oxygen atmosphere, followed by quantification through gas chromatography with a CHNS Thermo scientific Flash 2000 elemental analyzer. From these results, the PVP was calculated. Chemical analysis of Au and Ti was done with inductively coupled plasma optical emission spectroscopy (ICP-OES) with an ICP-OES Perkin-Elmer, model Optima 4300DV. The samples were previously digested with a mixture of concentrated HNO_3_, HCl, and HF in a Milestone Ultraware microwave. In all the cases, the mass analyzed was weighed with a microbalance MX5 Mettler Toledo. Samples were carried out in triplicate. The percentage of Au and TiO_2_ was calculated from these results. Infrared (IR) analysis was carried out in a FTIR Perkin-Elmer Spectrum One spectrometer, in ATR mode, between 450 and 4000 cm^−1^ energy range. Simultaneous thermogravimetric analysis (TGA)-differential scanning calorimetry/differential thermal analysis (heat flow DSC/DTA) was conducted in a NETZSCH-STA 449 F1 Jupiter equipment, from room temperature to 700°C in air. Scanning electron microscopy (SEM) images of the samples supported in filter paper were obtained at low vacuum mode (50 Pa) with a SEM QUANTA FEI 200, with a voltage of 15 kV.

### Preparation of the photocatalysts

Four milli grams of each synthesis (A and B), as-synthesized and calcined (at 400, 450, 550, and 600°C), were weighed in an Eppendorf. Then, 200 μL of ethanol were added and the samples were sonicated for 20 min. Filter paper from the laboratory (from Albet, pore size 35-40 μm, 80 g·m^−2^, thickness 0.18 mm), used as support, was cut in round-shapes and weighed. Then, the filter paper was impregnated with the NPs solution, by pouring each time 20 μL and evaporating the ethanol in an oven at 50°C. Only the center of the paper was impregnated, corresponding to the inner diameter of the photocatalytic reactor used for the tests. Once dry and at room temperature, the photocatalytic paper was weighted to know the real amount of material added.

### Photocatalytic activity

**Figure 2:**
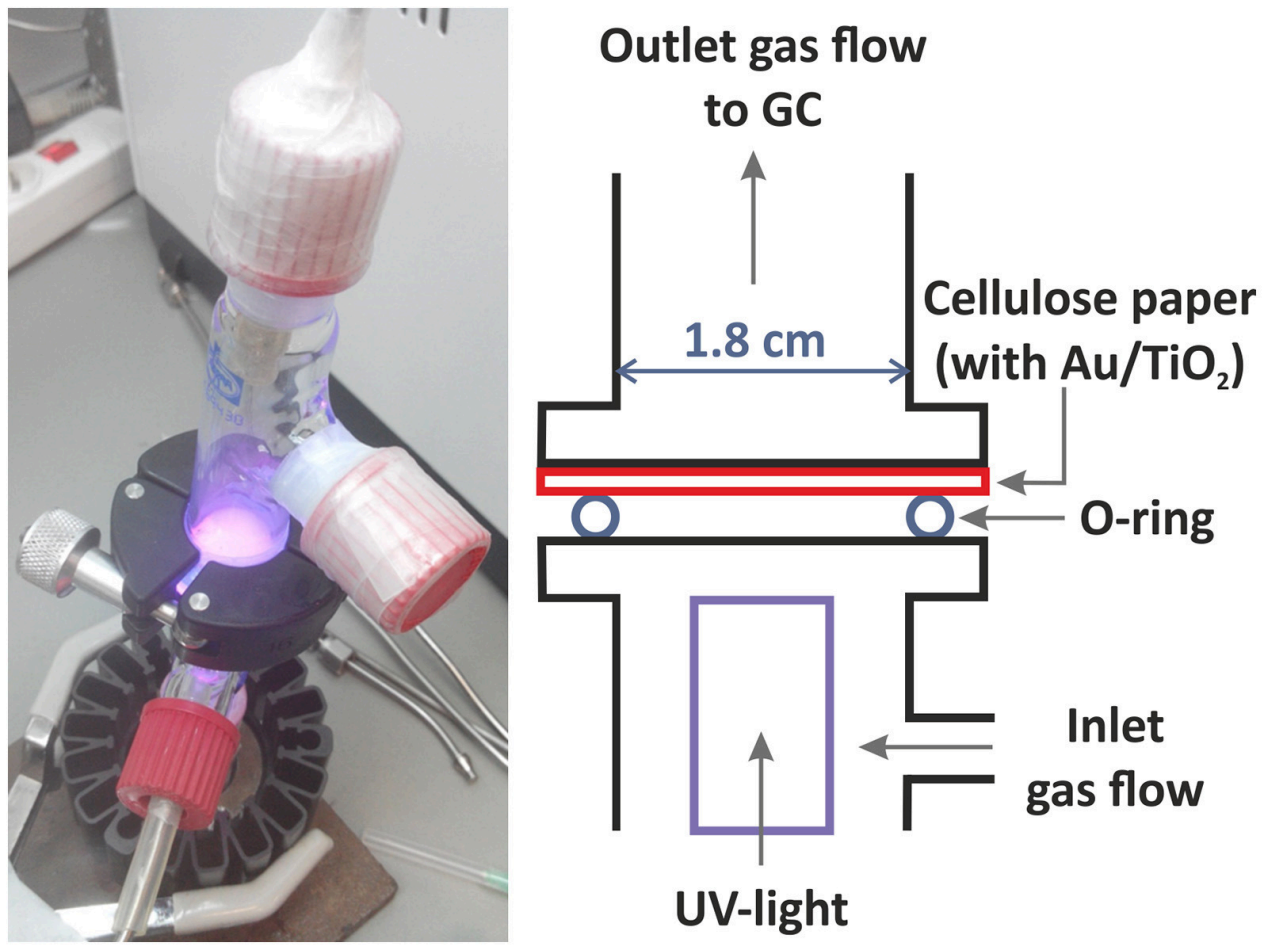
Image and scheme of the photoreactor. The UV-light LED irradiates the sample from below.

Photoreactions were carried out in gas phase at room temperature and atmospheric pressure in continuous mode in a tubular glass reactor previously described (Aguiló et al., 2017; Molins et al., 2017). In a typical experiment, the impregnated filter paper is placed between the two parts of the photocatalytic reactor, upside-down (the impregnated photocatalyst facing downwards) on top of the O-ring on the center the reactor, over the UV LED (Figure 2). The junction is tightly sealed with parafilm and a screw sealing ring. A saturated Ar gas stream with a water:ethanol vapor mixture (90:10 ratio on a molar basis) was introduced into the photoreactor by bubbling dry Ar gas at a flow rate of 20 mL/min through a saturator (Dreschel bottle) containing a liquid mixture of 87.5g of H_2_O and 9.92g of ethanol. The photoreactor effluent was monitored on-line every 4 min by gas chromatography (GC) (Agilent 3000 A MicroGC) using three columns: MS 5 Å, Plot U and Stabilwax. The LED UV-light source (from SACOPA, S.A.U.) consisted of four LEDs at 365 ± 5 nm and a synthetic quartz glass cylindrical lens that transmits the light to the photocatalyst. The UV-light source is located at the bottom part of the reactor, irradiating the filter paper from below, at a distance of 1 cm. Light irradiation is measured directly with a UV-A radiation monitor from Solar Light Co. and is 81.7 ± 0.5 mW·cm^−2^. In this set of experiments, we wanted to evaluate the photocatalytic activity of different catalysts under the same experimental conditions, so we did not want to be limited by the amount of irradiation. We worked with excess UVA light (365 nm) to not be limited by this factor. The irradiation area is quite small and is placed very near the light (<1 cm). This involves a low apparent quantum efficiency (AQE) achieved, since it represents the efficiency of the irradiated light to form hydrogen. The AQE value calculated in the maximum rate of hydrogen production (sample A at 450°C), is equal to AQE = 0.45% (see Supplementary Material for details of AQE calculation). At the beginning of each experiment, the UV light is off, and the reaction system is purged by entering 20 mL/min of saturated Ar gas with the water-ethanol vapor mixture, to remove the maximum possible oxygen (O_2_) in the line, up to reaching an O_2_ stable value. During this time, the GC chromatograms indicate a reduction of the oxygen content, which decreases down to very low values (<0.001%). Due to the intrinsic limitations of the system, it is not possible to completely remove the oxygen inside the reaction system. After 30 min, the UV light is turned on and we monitor all photoreaction products during ca. 20–40 min by GC. Control experiments were carried out with only the filter paper support and no photoactivity was measured. Two measurements were made for each as-synthesized sample (A and B) with excellent reproducibility. Experiments with bare TiO_2_ NP and Au NP were also performed.

## Results and discussion

The TiO_2_/Au nanoparticles were synthesized, in <1 h, through a 2-step microwave-assisted synthesis (Figure 1). In the first step, the TiO_2_ NPs are formed by sol-gel synthesis in a microwave oven using titanium butoxide (TBOT) as precursor, benzyl alcohol (BA) as solvent, and polyvinylpyrrolidone (PVP) as stabilizer. The PVP capped-TiO_2_ NPs with an average size of 9 ± 2 nm are crystalline presenting solely the anatase phase. In the second step, two different syntheses, labeled A and B, are performed to obtain two different sizes of Au NPs (see A and B in Figure 1) in order to evaluate their different photocatalytic behavior. In the presence of the pre-formed anatase nanoparticles, the second step is a polyol-synthesis for the formation of Au NPs using HAuCl_4_·3H_2_O as precursor, ethylene glycol (EG) as solvent and PVP as reducing and capping agent. The TiO_2_ NPs, dispersed in the reaction media, serve as nucleation sites for the Au NPs. In this second step, the amount of PVP is used to tune the size of the Au NPs as represented in Figure 1, confirming the significant role of PVP in the formation of anisotropic nanostructures (Yu et al., 2015). While small PVP/Au precursor molar ratio (0.625:1) lead to large Au NPs with anisotropic shapes including triangles, an increase of PVP/Au precursor molar ratio (3.75:1) yields smaller and rounded Au NPs, as was also observed in the SPIONs/Au system (Yu et al., 2015). The synthesis presented here reinforces the potential of microwave irradiation to fabricate multimaterial NPs in a short time and in a easily scalable process (Gonzalez-Moragas et al., 2015; Yu et al., 2015; Hachtel et al., 2016). Finally, we also prepared Au NPs using the same protocol of “Synthesis A” but without the PVP capped-TiO_2_ NPs, obtaining spherical Au NPs of 9 nm mean size, as previously shown by Yu et al. (2015).

**Figure 3:**
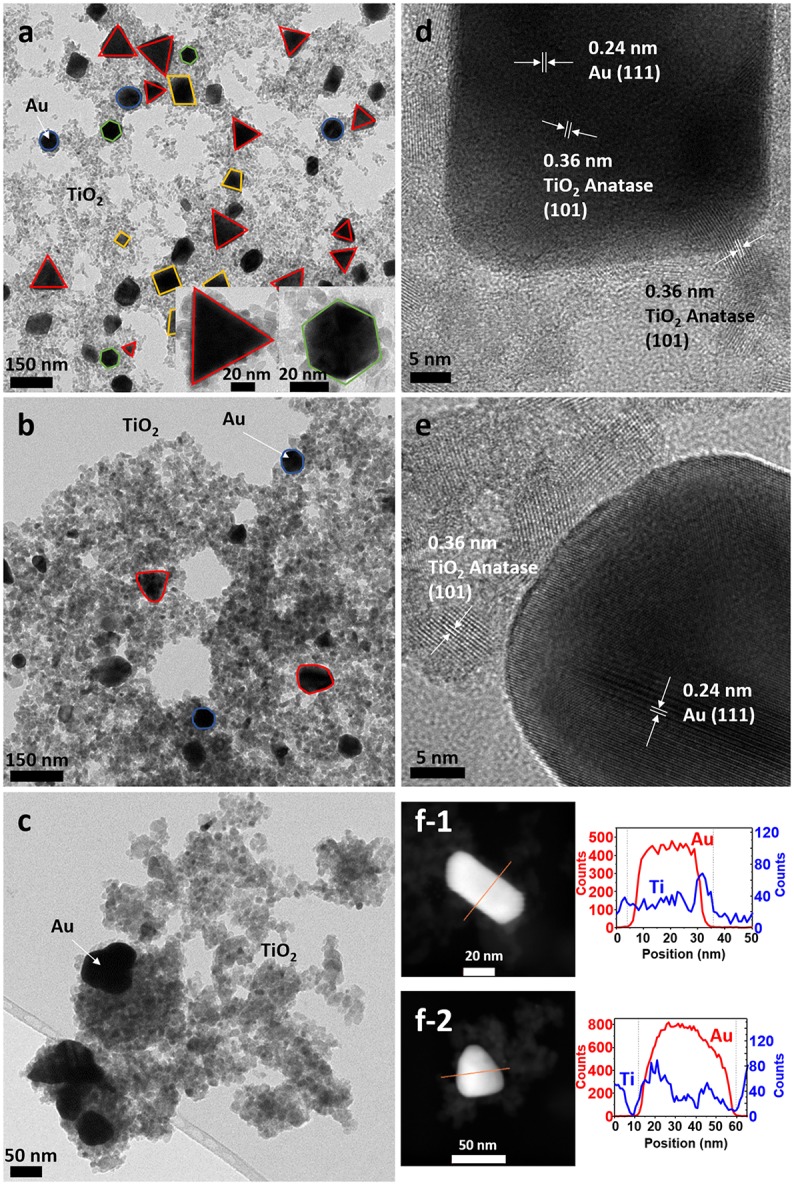
TEM images of sample A **(a)** as-synthesized (NC: not calcined), **(b)** calcined at 450°C and **(c)** calcined at 600°C. HRTEM images of sample A **(d)** as-synthesized, **(e)** calcined at 450°C, **(f)** STEM of sample A of **(f-1)** as-synthesized and **(f-2)** calcined at 450°C, with element profiles.

**Figure 4:**
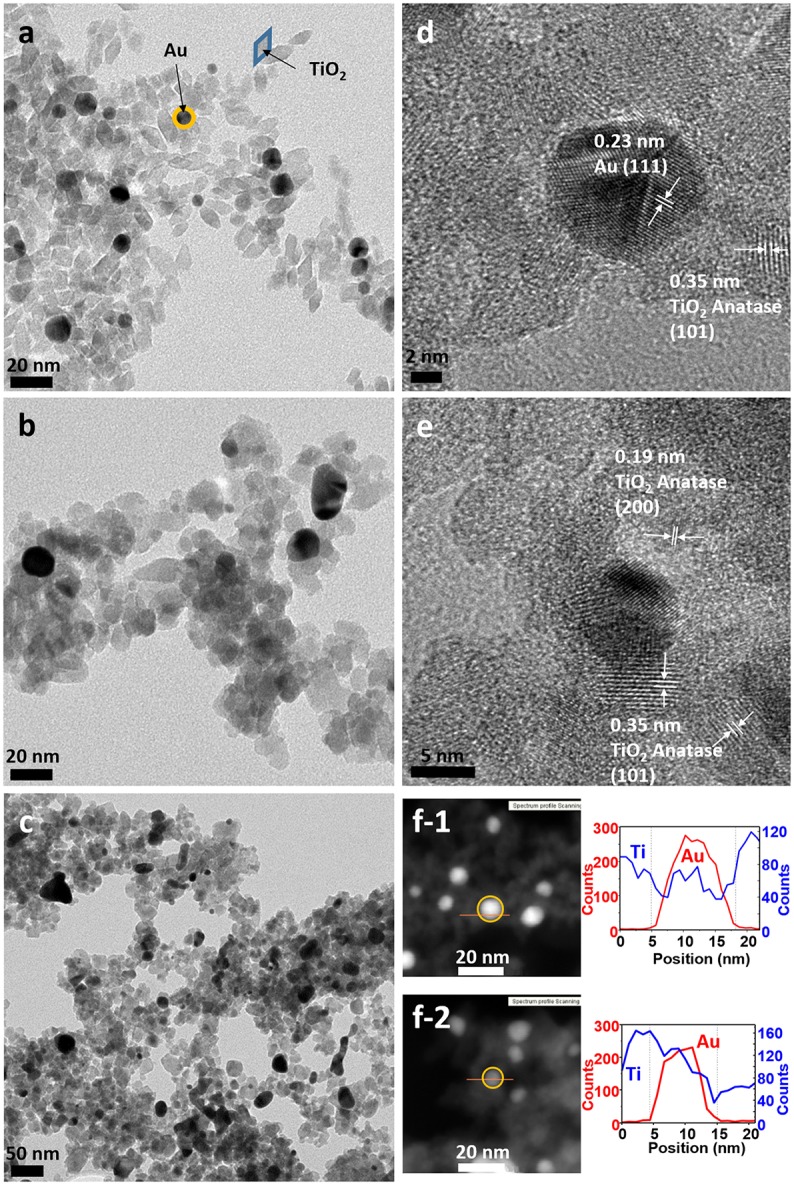
TEM images of sample B **(a)** as-synthesized (NC: not calcined), **(b)** calcined at 450°C and **(c)** calcined at 600°C. HRTEM images of sample B **(d)** as-synthesized, **(e)** calcined at 450°C, **(f)** STEM of sample B of **(f-1)** as-synthesized and **(f-2)** calcined at 450°C, with element profiles.

TEM micrographs confirmed the successful formation of the nanoparticles and the differences in particle size and shape of both syntheses. Synthesis A (Figure 3a) with Au NPs (average size > 50 nm) of different shapes, including nanotriangles (22%), nanocubes (40%), nanospheres (31%), nanohexagons (5%), and nanopentagons (2%), which are randomly decorated with small TiO_2_ NPs (9 ± 2 nm in size). On the other hand, synthesis B (Figure 4a) involves the formation of much smaller Au nanospheres (8 ± 2 nm), very similar in size as that of the TiO_2_ NPs (9 ± 2 nm), although different in morphology (TiO_2_ NPs have a rhombus shape). Moreover, due to the similar sizes, the Au NPs cannot be decorated with TiO_2_ NPs, but they are always surrounded by them: in all the analyzed images, no isolated Au NPs are found.

Electron diffraction patterns of the as-synthesized samples (Supplementary Figures 1a,d in the Supplementary Material) indicate that titanium dioxide is anatase, which is the most photocatalytic active phase. This phase is already formed during the microwave synthesis process, without a further annealing step needed. The electron diffraction patterns also confirm the presence of metallic gold in the samples. Samples were calcined at different temperatures to remove the organic PVP that keeps the two materials apart, in order to increase the contact between the two inorganic phases and enhance the electron transfer between them (Azhari and Diab, 1998). Calcination has larger effect on the Au than on the TiO_2_ NPs. Au NPs of sample A calcined at 450°C show more rounded corners, especially for the triangles and the cubes (Figure 3b). These changes are more severe at 600°C (Figure 3c) when the gold NPs start to melt and fuse with each other, forming elongated-rounded shapes and resulting in a particle size increase. The average particle size of sample A increases around 7 nm (up to 59 ± 21 nm), whereas that of sample B increases much more (28 ± 9 nm) (Figures 4b,c). Regarding TiO_2_ NP, they maintain their size up to 450°C whereas after being calcined at 600°C, they increase up to 13 ± 4 nm and 17 ± 5 nm for synthesis A and B, respectively. However, upon calcination at the different temperatures, TiO_2_ remains as anatase (Supplementary Figures 1a–c, d–f). The increase of particle size with calcination temperature and the particle size distributions are illustrated in Supplementary Table 1, Supplementary Figures 2, 3. Annealing at 450°C removes almost totally the PVP, largely increasing the intimate contact between Au and TiO_2_ NPs, as observed by TGA analysis and IR analysis, included in Supplementary Figures 4, 5.

By HRTEM we can image the interplanar spacing of TiO_2_ and gold. A lattice spacing of 0.35 ± 0.01 nm, measured in both samples, matches the (101) lattice plane for TiO_2_ anatase. In sample B calcined at 450°C, an additional spacing of 0.19 ± 0.01 nm is detected, that is assigned to the (200) anatase plane. The 0.23 ± 0.01 nm lattice spacing of Au NPs belongs to the (111) plane for metallic Au (Figures 3d,e, 4d,e). Moreover, HRTEM also highlights the homogeneous covering and contact between Au and TiO_2_. In sample A, we can see that the large Au NPs are covered by the TiO_2_ NPs, since some anatase planes can be identified on top of the Au NPs (Figure 3d) and after the PVP is removed we can see an intimate contact between both materials (Figure 3e). The coverage of Au NPs by TiO_2_ is also confirmed by HAADF STEM (Figure 3f), an element profile shows that Ti is identified on the top of the Au particles. In sample B with smaller Au NPs, these are always surrounded by similar-size TiO_2_ NPs (Figure 4f). We could not find Au NPs that were not in contact by at least one TiO_2_ NP. The element profile obtained by HAADF STEM also indicates the presence of Ti where there is the Au NP in this sample.

TEM micrographs also show an excess of TiO_2_ NPs that are not in contact with Au NPs, which will have little or no effect on the photocatalytic activity. To remove the excess of TiO_2_, both samples were further purified by centrifugation as explained in the experimental section. TEM micrographs of the purified samples (Supplementary Figures 6a–e) show that the excess of TiO_2_ was partly removed for sample A. Unfortunately, this purification method was unsuccessful for sample B, due to the similarity of particle size between TiO_2_ and Au NPs. Photocatalytic activity for the purified A and B samples (labeled as A^*^ and B^*^) will also be reported below.

The amount of Au and TiO_2_ on the samples has been quantified by ICP-OES. The amount of PVP has been indirectly calculated by elemental analysis of CHNS (carbon, hydrogen, nitrogen and sulfur), considering PVP as (C_6_H_9_NO)_n_,. The ICP-OES results compared well with those obtained from the thermogravimetric analysis (TGA) (Supplementary Figure 4). Despite using the same initial amount of gold precursor, sample A (with 20 ± 2 wt%) has nearly double gold content than sample B (with 11 ± 2 wt%), whereas the amount of TiO_2_ is similar in both samples (49 ± 7 wt% and 51 ± 7 wt%). From the elemental analysis, it was interfered that the amount of PVP was 31 ± 5 wt% in sample A, and 38 ± 5 wt% in sample B. Infrared (IR) analysis confirmed the presence of PVP in the TiO_2_ NPs and in A and B samples, and proved the removal of the PVP on the calcined samples (Supplementary Figure 5).

The purification of the samples from an excess of TiO_2_ leads also to a decrease of the PVP content, from 31 to 5 wt% in sample A^*^, and from 38 to 10 wt% in sample B^*^, and obviously to a remarkable increase of the Au content, especially in sample A^*^, from 20 to 67 wt%, whereas in sample B^*^ from 11 to 20 wt%. Supplementary Figure 7, depicts the distribution of the Au/TiO_2_/PVP for all samples.

**Figure 5:**
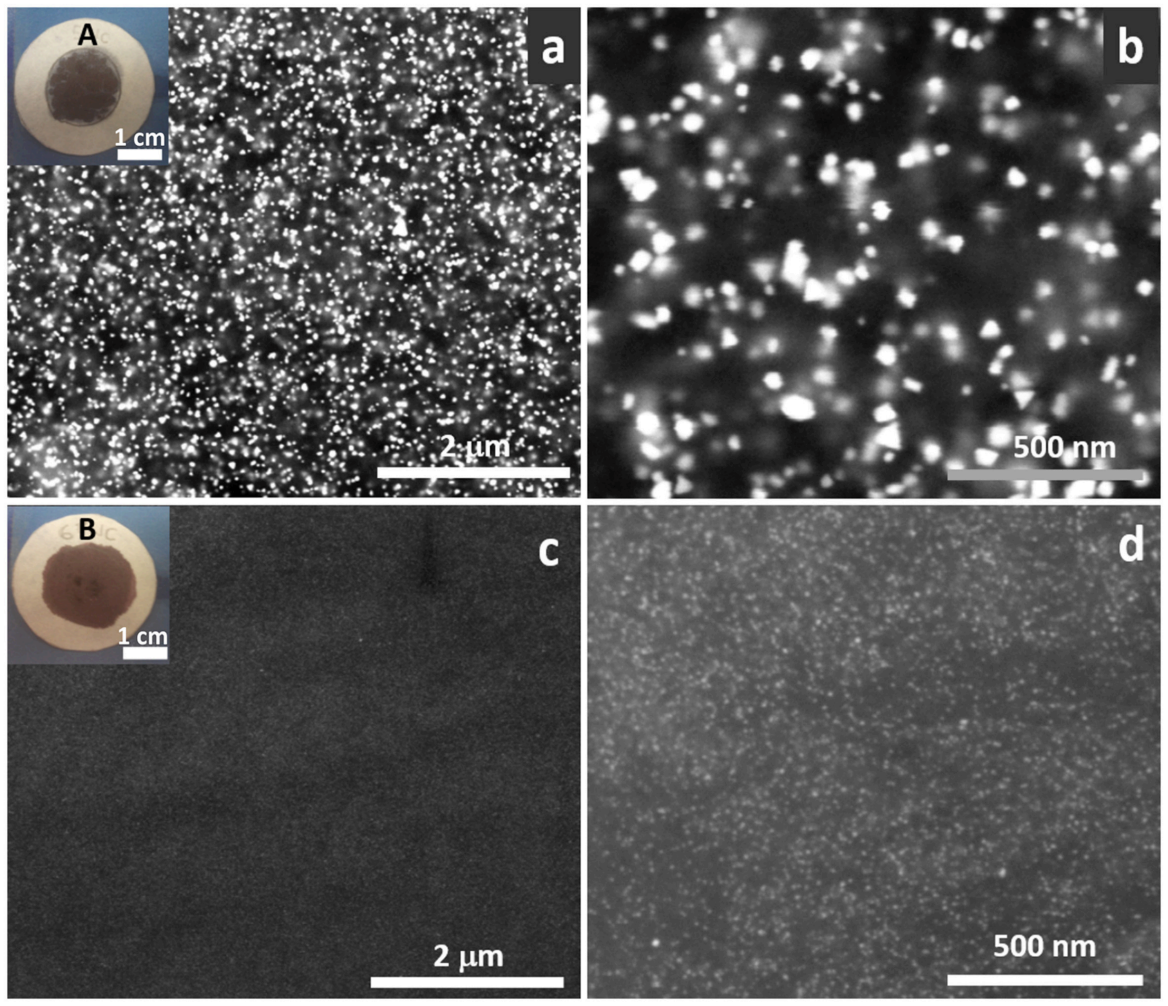
Backscattered SEM images of sample A **(a,b)** and B **(c,d)** on top of the filter paper at 50,000 x **(a,c)** and 200,000 x **(b,d)**. Bright dots correspond to Au NPs. Insets correspond to photographs of the materials deposited on filter paper **(a)** sample A, **(c)** sample B.

To evaluate the photocatalytic activity of these systems, the TiO_2_/Au NPs solutions were deposited on top of ordinary laboratory filter paper. The amount of deposited photocatalyst was set to 4 mg, in order to achieve an optimal photocatalyst loading value of ca. 1.2 mg/cm^2^ previously reported by Castedo et al. (2016). The insets in Figure 5 show the materials corresponding to the as-synthesized samples A and B. The rest of the used samples are shown in Supplementary Figure 8. SEM images were obtained of the as-prepared photocatalytic papers. Figure 5 shows that the distribution of the nanostructures is very homogenous in both sample A (Figures 5a,b) and B (Figures 5c,d). This is better confirmed by Au NPs (shown as bright spots) evenly dispersed in all the area. In sample A at 200,000 x (Figure 5b) the different shapes of the particles can be differentiated (nanotriangles, squares, spheres…).

**Figure 6:**
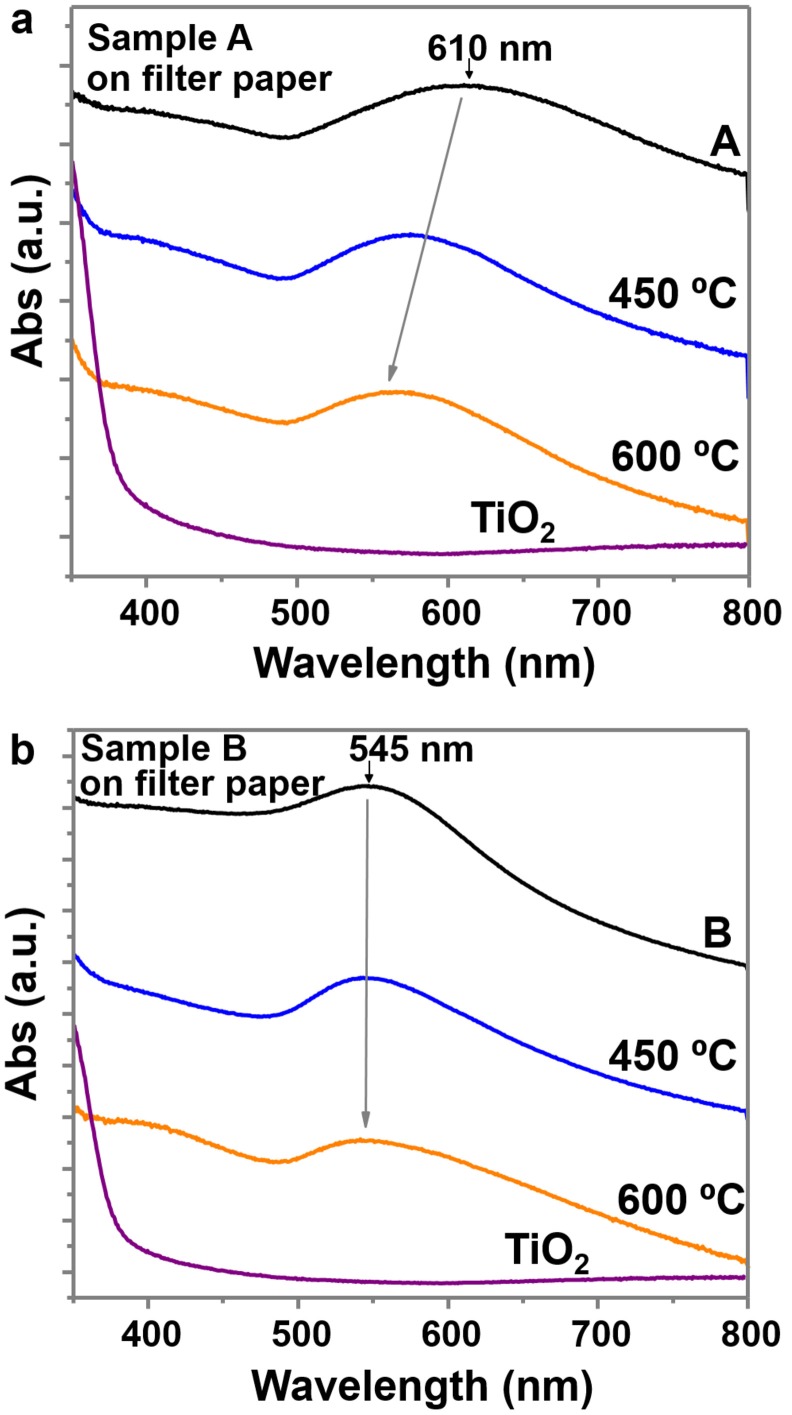
UV-Vis spectra between 350 and 800 nm wavelength of **(a)** sample A and **(b)** sample B, on filter paper and at different calcined temperatures. Both plots include the UV-Vis spectra of TiO_2_ NPs on filter paper (before adding the gold precursor).

UV-Vis absorption spectra collected between 350 and 800 nm were recorded on these materials (Figure 6). Both samples show the gold plasmon absorption peak, with absorption of sample A at longer wavelengths (610 nm) than sample B (545 nm), due to the larger particle size and anisotropic shapes (Scarabelli et al., 2014; Yu et al., 2015). The UV-Vis spectra of the purified samples (A^*^, B^*^) maintained the gold absorption peaks at the same wavelength and the same TiO_2_ absorption at the UV region, indicating the presence of both elements (Supplementary Figures 9a,b). The UV-Vis spectra of the successive supernatants of the different purification steps (Supplementary Figure 9c) and the evolution of sample A (Supplementary Figure 9d) confirm the removal of TiO_2_ and the retention of most Au NPs. Some images of the supernatants and of the final purified samples are also provided (Supplementary Figure 9e). Calcination has an effect on the absorption peak of sample A, which decreases to values of 565 nm due to the modification of the particle shape and lose of anisotropy (rounded edges; Pelaz et al., 2012). However, the absorption peak of sample B is not modified. The TiO_2_ sample clearly shows an increase of absorption at the UV region, while in the other samples this increase is not so evident, probably due to the lower concentration of TiO_2_ on sample A and B.

All the synthesized photocatalysts were studied in the photoproduction of H_2_ under dynamic conditions at atmospheric pressure and room temperature, introducing a saturated Ar gas stream with a water:ethanol vapor mixture at a flow rate of 20 mL/min through a tubular photoreactor. In addition, blank experiments with bare TiO_2_ NPs and Au NPs were also carried out for comparison. The gaseous reactants (saturated Ar with water-ethanol mixture 9:1 molar, GHSV~26,000 h^−1^) enter the reactor from the lower part, passes through a cellulose paper loaded with the TiO_2_/Au catalyst and exits the reactor from the top (see Figure 2). The outlet of the photoreactor was continuously monitored by GC. H_2_ was produced using the two investigated catalysts (A and B) in both the as-synthesized and the calcined forms. H_2_ was also produced using bare TiO_2_ NPs. The production of H_2_ remained stable with time (from ~7 to 40 min) after an initial transient period and stabilization of the reaction. Acetaldehyde is the only byproduct detected in all the experiments using TiO_2_ based photocatalysts. The use of Au NPs in the absence of TiO_2_ NPs did not yield H_2_ production under our experimental conditions.

**Figure 7:**
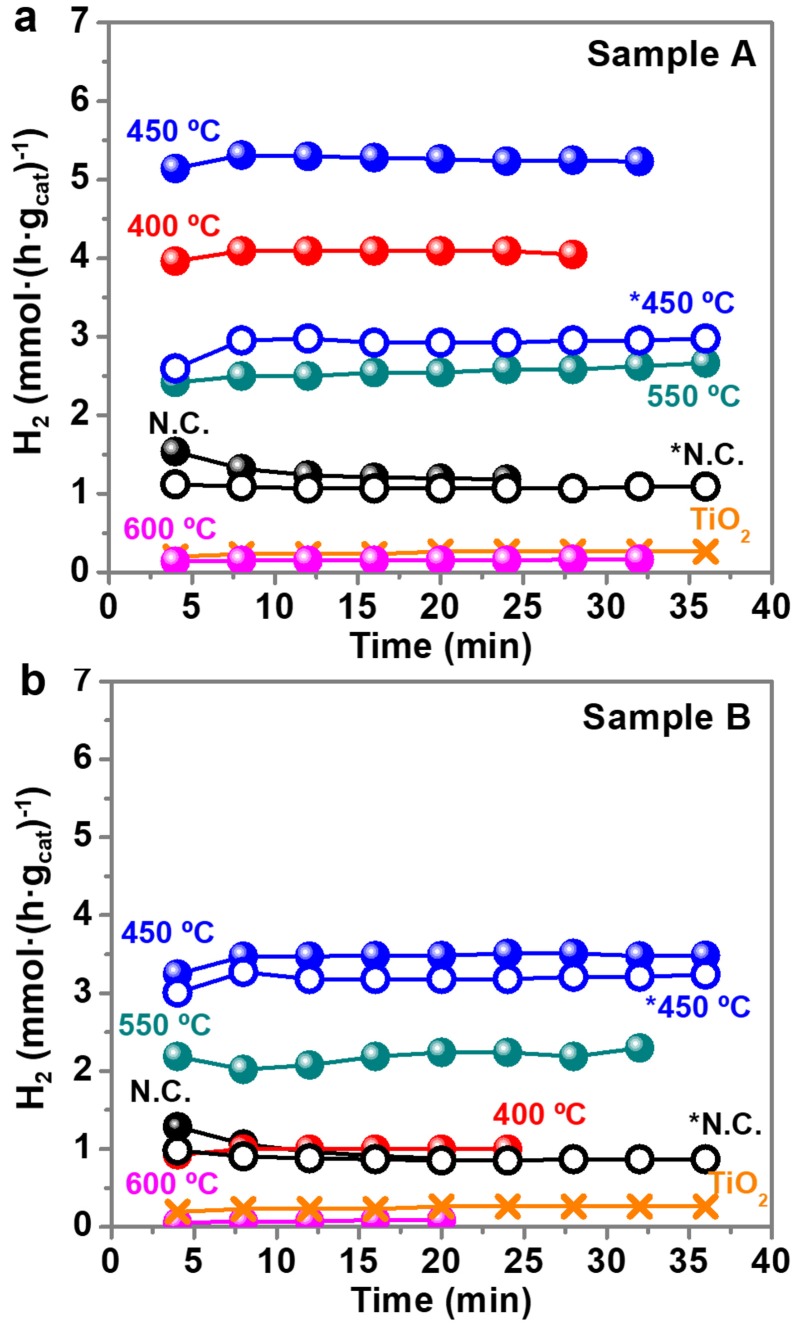
Hydrogen production (mmol·(h·g_cat_)^−1^) with time for sample A **(a)** and sample B **(b)**. Notation: N.C: not calcined; * purified samples. Time = 0 min (UV light on), Time = 4 min (first GC measure with UV light on). The UV light remained on during all the collected data points.

Figure 7 shows the UV light driven hydrogen production of sample A (Figure 7a) and sample B (Figure 7b) calcined at different temperatures and normalized per mass of catalyst. The mass of PVP is non-relevant in the total mass of the catalyst because it is removed with the calcination, except for the non-calcined sample that contains PVP. The percentage of Au and TiO_2_ in the calcined samples is higher than in the non-calcined sample, because they are all prepared from the same initial weight fraction of PVP:Au:TiO_2_ (see Supplementary Figure 7). The UV light is turned on at *t* = 0 min, and the first result from the GC under UV light is obtained at *t* = 4 min, as shown in Figure 7. The time profiles demonstrate that the stable and constant amounts of hydrogen are produced over the photocatalyst under UV light irradiation during all the experiments. A blank experiment using bare anatase TiO_2_ in the absence of Au NPs presents a low activity, producing 0.26 mmol H_2_·${\text{g}}_{\text{cat}}^{-1}\cdot$h^−1^. However, when the surface of Au NPs is decorated with non-calcined TiO_2_ NPs, the hydrogen evolution efficiency enhances significantly, 4.5 times for sample A (1.2 mmol H_2_·${\text{g}}_{\text{cat}}^{-1}\cdot$h^−1^) and 3.3 times for sample B (0.9 mmol H_2_·${\text{g}}_{\text{cat}}^{-1}\cdot$h^−1^). Murdoch et al. (2011) affirmed that the role of Au NPs is crucial in the water splitting reaction using TiO_2_ semiconductor materials, as some steps to achieve the photo-production of hydrogen by Equation (1) did not take place in its absence. As expected, the calcination treatments further enhance the hydrogen production. As shown in Figure 7, the optimal calcination temperature to reach the best photocatalytic performance for H_2_ evolution is 450°C. Calcination results in a more intimate contact between TiO_2_ NPs and Au NPs. However, calcination temperatures above >550°C detrimentally diminish the number of active sites on the surface of the photocatalyst, probably by decreasing the surface area, since the particle size increase and particles aggregate and fuse together. This can also reduce the light penetration, preventing the activation of TiO_2_. At 600°C, remarkably low values of hydrogen production, similar to bare TiO_2_, are obtained.

**Figure 8:**
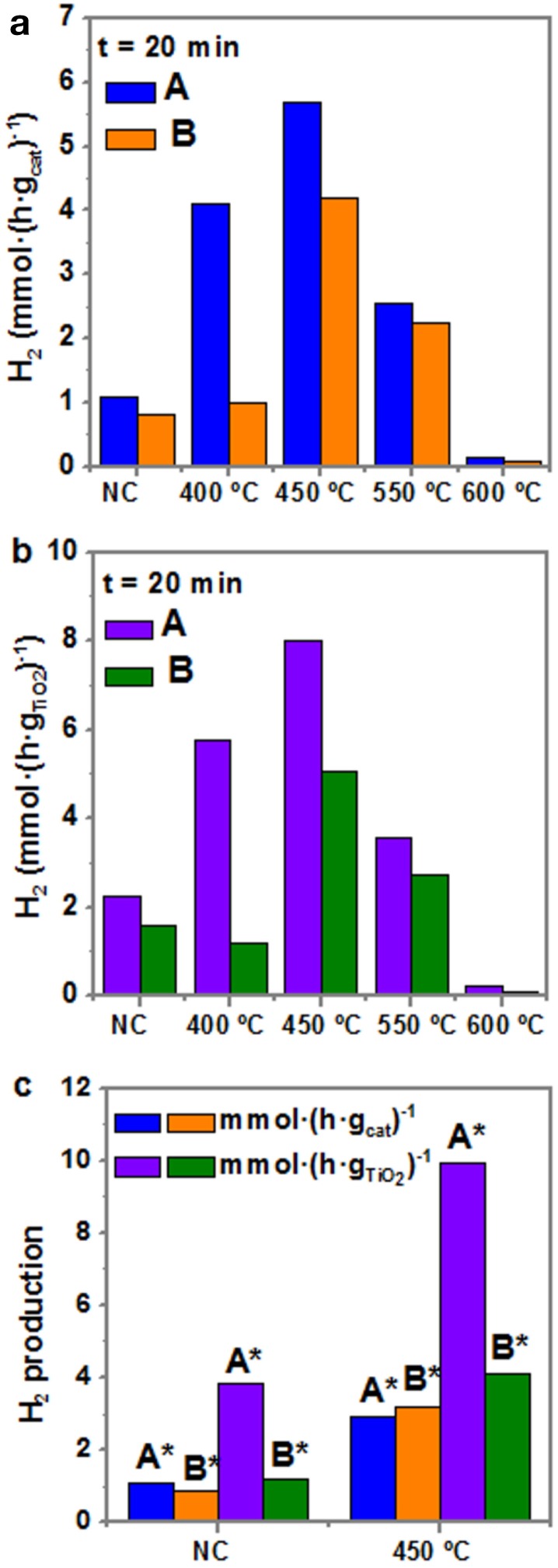
Hydrogen production rate after 20 min of photocatalytic reaction under UV irradiation of samples A and B at different thermal treatments **(a)** per mass of catalyst, and **(b)** per mass of TiO_2_. **(c)** Hydrogen production of purified samples after 20 min of reaction, per mass of catalyst and mass of TiO_2_ NC and at 450°C. Notation: N.C, not calcined; * purified samples.

Figure 8a depicts the photocatalytic production of H_2_ (mmol·${\text{g}}_{\text{cat}}^{-1}\cdot$h^−1^) after 20 min of reaction for both samples A and B as-synthesized, i.e., not calcined, and at the different calcination temperatures (from 400 to 600°C). As observed in Figure 8a, the photocatalytic activity of the as-synthesized sample is around 1 mmol·g^−1^·h^−1^, being higher for sample A than for sample B (1.2 > 0.9). For the samples treated at 400°C, the activity increases up to 4 times for sample A (up to 4 mmol·g^−1^·h^−1^), whereas it remains nearly the same for sample B. However, the highest activity is reported when the samples are calcined at 450°C: up to ~5.3 mmol·g^−1^·h^−1^ for sample A and 3.5 mmol·g^−1^·h^−1^ for sample B. From 550°C the activity decreases and at 600°C it is even less than for the as-obtained samples. To achieve a better comparison of the photocatalytic performance of both samples A and B with the previously reported data in the literature, Figure 8b presents H_2_ production normalized per mass of TiO_2_. The overall trend is the same, with higher values of H_2_ production rates, reaching a maximum of 7.5 mmol·g^−1^·h^−1^ in sample A at 450°C. Regarding the purified samples A^*^ and B^*^, their photocatalytic production of H_2_ of normalized per mass of catalyst and per mass of TiO_2_ is shown in Figure 8c. Interestingly, the production of hydrogen for calcined purified A^*^ presents larger values (> 10 mmol·${\text{g}}_{\text{TiO}2}^{-1}\cdot$h^−1^) than that of calcined sample A, because the excess of TiO_2_ has been removed and, consequently, the Au:TiO_2_ ratio increases. These results clearly indicate the need of intimate contact within TiO_2_ and Au in order to allow electron transfer and the role of Au to act as an electron reservoir.

Considering all the obtained results, sample A has more amount of gold per gram of catalyst than sample B. However, the Au NPs of sample A are bigger with a total specific surface area twenty-fold smaller than for sample B (0.4 ${\text{m}}_{\text{Au}}^{2}$/g for sample A compared to 8.4 ${\text{m}}_{\text{Au}}^{2}$/g for sample B). Nevertheless, we can observe from the TEM images (Figure 3 and Supplementary Figure 6) that the Au NPs of sample A are completely breaded by TiO_2_ NP. We thus argue that in sample A there is much bigger interface area between gold and titania, yielding an enhanced photocatalytic activity. The strong contact between Au and TiO_2_ NP is indispensable, as clearly observed by Haruta (2002), who found very different activities between Au hemispherical NPs with their flat planes strongly attached to the TiO_2_ support, compared to spherical particles simply loaded on the TiO_2_. However, the exposed surface area of gold is not directly associated with the activity of TiO_2_/Au toward hydrogen production (Bamwenda et al., 1995). Bamwenda et al. (1995) reported that the essential reaction steps occurred on the semiconductor surface, and that the microinterfaces between Au and TiO_2_ could also play a role as active sites. Hence, one decisive factor is the contact area between the noble metal and the semiconductor, which is where the Schottky junctions are formed, and enhance the separation of photo-excited electrons and holes. In addition, as the metal acts as a “fast lane” to transfer the electrons injected from the TiO_2_ to the active sites, where they are consumed in the hydrogen generation reaction (2${\text{H}}_{\left(\text{ads}\right)}^{+}$ + 2e^−^ → H_2_), it is necessary that they can move without encountering any barriers. The stronger and more expanded contact area between TiO_2_ and Au NPs explains why the H_2_ production is larger for TiO_2_ NPs supported on large Au NPs (sample A) than for TiO_2_ NPs in close contact to Au NPs of similar sizes (sample B).

Our results are consistent with the reaction scheme involved in the photo-production of hydrogen from ethanol over Au/TiO_2_ and previously proposed by Murdoch et al. (2011). Equation (1) is a multistep reaction. Briefly, an ethanol molecule is dissociatively adsorbed on the photocatalyst surface to form an ethoxide and a hydrogen ion (as a surface hydroxyl), as follows

(2) $$\begin{array}{rc} {\text{CH}}_{3}\text{C}{\text{H}}_{2}\text{OH} & +{\text{T}{\text{i}}^{4+}}_{\left(\text{surface}\right)}-{\text{O}}_{\left(\text{surface}\right)}^{2-}\\ \;\to \text{C}{\text{H}}_{3}\text{C}{\text{H}}_{2}\text{O}-{\text{T}{\text{i}}^{4+}}_{\left(\text{surface}\right)}+\text{O}{\text{H}}_{\left(\text{adsorved}\right)} \end{array}$$

Then, electron-hole pairs are photogenerated on the photocatalyst surface [Equation (3)], ethoxides inject two electrons into the valence band and acetaldehyde is produced [Equations (4, 5)], and two hydrogen ions are reduced to a hydrogen molecule by two electrons from the conduction band [Equation (6)].

(3) $$\begin{array}{r} \text{Ti}{\text{O}}_{2}+\text{UV}\to {\text{e}}^{-}+{\text{h}}^{+} \end{array}$$

(4) $$\begin{array}{rcl} {\text{C}{\text{H}}_{3}\text{C}{\text{H}}_{2}\text{O}-\text{T}{\text{i}}^{4+}}_{\left(\text{surface}\right)}+{\text{h}}^{+} & + & {\text{O}}_{\left(\text{surface}\right)}\\ \to {\text{C}{\text{H}}_{3}\text{C}{\text{H}}^{•}\text{OT}{\text{i}}^{4+}}_{\left(\text{surface}\right)}+\text{O}{\text{H}}_{\left(\text{adsorbed}\right)} \end{array}$$

(5) $$\begin{array}{r} \text{C}{\text{H}}_{3}\text{C}{\text{H}}^{•}\text{OT}{\text{i}}_{\left(\text{surface}\right)}^{4+}+{\text{h}}^{+}\to \text{C}{\text{H}}_{3}\text{CH}{\text{O}}_{\left(\text{g}\right)}+\text{T}{\text{i}}_{\left(\text{surface}\right)}^{4+} \end{array}$$

(6) $$\begin{array}{r} 2\text{O}{\text{H}}_{\left(\text{adsorbed}\right)}+2{\text{e}}^{-}\to 2{\text{O}}_{\left(\text{surface}\right)}^{2-}+{\text{H}}_{2} \end{array}$$

The presence of water is important to avoid the blockage of the active sites of the photocatalyst by adsorption of acetaldehyde molecules.

This study also demonstrates that H_2_ can be obtained from water-ethanol mixtures in gas-phase with a photocatalyst composed of large Au NPs, and small TiO_2_ NPs, containing high amounts of gold (> 10–20 wt%). Hence, the photogeneration of H_2_ is not only restricted to small Au particle size and low gold percentages (<2 wt%), as the majority of studies have reported so far (Table 1). The studies reported in Table 1 are, in general, with a small Au loading (1–2 wt%) and with small Au NPs particle size (1–10 nm). Although the studies that use methanol as hole scavenger achieve higher values of H_2_ production, the reported hydrogen activity is of the same order of magnitude as this present study (7-17 mmol·g^−1^·h^−1^ compared to 5.3 mmol·g^−1^·h^−1^ obtained for the TiO_2_/Au sample A calcined at 450°C).

**Table 1:** Reported gas-phase photocatalytic performance in recent catalytic systems formed by Au/TiO_2_ NPs.

**Material**	**Au loading (wt%)**	**Particle size (Au/TiO_2_) (nm)**	**Hole scavenger**	**Ratio water:hole scavenger**	**Wavelength and power irrad. lamp**	**Max. rate of H_2_ (mmol g^−1^ h^−1^)**	**References**
Au/TiO_2_ Anatase (A)^[a]^	20	30–70 (52 av.)/9	Ethanol	90:10 (molar)	365 nm 81.7 mW/cm^2^	5.3 QE^[d]^ = 0.45%	This study
Au/TiO_2_ Anatase (B)^[a]^	11	8/9	Ethanol	90:10 (molar)	365 nm 81.7 mW/cm^2^	3.5	This study
Au/TiO_2_ Anatase (A*)^[a]^	67	30–70 (52 av.)/9	Ethanol	90:10 (molar)	365 nm 81.7 mW/cm^2^	2.9	This study
Au/TiO_2_ Anatase (B*)^[a]^	20	8/9	Ethanol	90:10 (molar)	365 nm 81.7 mW/cm^2^	3.2	This study
Au/TiO_2_ P90^[b]^	1.8	4/P90 size^[c]^ (15–20)	Ethanol	90:10 (molar)	365 nm 1.5 mW/cm^2^	≈ 5 QE = 9.2%	Castedo et al., 2016
Au/TiO_2_	1	3.8/30–40	Ethanol	0:100	365 nm 2.6 mW/cm^2^	≈ 2.75^[e]^ (mmol·g^−1^·h^−1^W^−1^)	Bonmatí et al., 2015
Au/TiO_2_ Anatase:rutil ≈ 93:7	1-1.5	3.9/20–40	Ethanol	0:100	365 nm 4 × 12 W	≈ 0.6^[e, f]^ QE = 20.8%	Taboada et al., 2014b
Au/TiO_2_ P25^[b]^	1	4–10/P25 size^[c]^ (20–25)	Methanol	94:6 (% v/v)	330-450 nm 250 W	≈ 17^[e]^	Dozzi et al., 2010
Au/TiO_2_ Anatase:rutil ≈ 93:7	1	1–6/5–10	Methanol	94:6 (% v/v)	330-450 nm 250 W	10.2 QE = 6.3%	Chiarello et al., 2009
Au/TiO_2_ P25^[b]^	1	2–3/20	Methanol	94:6 (% v/v)	330-450 nm 250 W	7	Chiarello et al., 2009

*[a] All the samples were calcined at 450°C. [b] P25 and P90 are commercial TiO_2_ made of a mixture of anatase and rutile phases, with a ratio anatase:rutile of 80:20 and 98:2, respectively (Siah et al., 2016). [c] P25 NP size is around 20-25 nm and P90 NP size is somewhat smaller, around 15-20 nm (Viswanathan and Raj, 2009; Siah et al., 2016). [d] UV irradiation technical details and Apparent Quantum Efficiency can be found at Materials and Methods and Supplementary Material. [e] Values taken from a plot. [f] Original values were in other units, and have been recalculated to mmol·g^−1^·h^−1^*.

## Conclusions

We have successfully developed a synthetic route to obtain two different sizes of Au/TiO_2_ NPs by a fast and simple two-step microwave-assisted synthesis (<1 h of overall synthesis time). Hereby, we have clearly exposed that microwave chemistry is a very efficient method to achieve complex nanostructures. While the larger Au NPs (*ca*. 50 nm) are breaded with the small titanium oxide NPs containing abundant interfacial contacts between gold and titania, the smaller Au NPs (*ca*. 10 nm) form dimers and trimers with the TiO_2_ NP of similar size and contain lesser contact points between the metal and the metal oxide.

The photocatalytic activity of the two Au/TiO_2_ nanostructures was evaluated in the photoproduction of hydrogen from gaseous water/ethanol mixtures at ambient temperature and pressure. We have shown that H_2_ production is accomplished with the two photocatalysts, both containing large co-catalyst fraction (~ 10–20 wt%). We thus conclude that in these cases the metal is not acting as recombination center for the photogenerated electrons and holes, as earlier postulated.

Our study also exposed that calcining at 450°C significantly facilitated charge transfer between the two materials, without compromising the catalyst surface and active sites and without affecting particle sizes. Importantly, the nanostructure with larger contact area between the metal and the semiconductor provided the best performance in terms of H_2_ production. Hence, the number of Schottky junctions is a decisive key parameter on the photocatalytic performance by enhancing the separation of photo-excited electrons and holes. Comparing our two systems, we determine that the later aspect has a more significant impact on the H_2_ production than the size or the load fraction of the metal co-catalyst.

Further work will be devoted to investigate the influence of the gold plasmon resonance absorption on the photocatalytic activity using solar light.

## Author contributions

AM-M and LS: Designed the experiments; AM-M, MT, and PS: Carried out the nanocomposites synthesis and characterization studies; AM-M and LS: Carried out the photocatalytic evaluations; JL and AR: Supervised the project. All authors wrote and reviewed the manuscript.

### Conflict of interest statement

The authors declare that the research was conducted in the absence of any commercial or financial relationships that could be construed as a potential conflict of interest.
