# Supplementary material for: Fast and Simple Microwave Synthesis of TiO2/Au Nanoparticles for Gas-Phase Photocatalytic Hydrogen Generation
Source: Front Chem. 2018 Apr 12;6:110. doi: 10.3389/fchem.2018.00110 (PMC5907367; doi:10.3389/fchem.2018.00110)
Supplement: Supplementary file 1 [file DataSheet1.docx]

Supplementary Material

Fast and simple microwave synthesis of TiO_2_/Au nanoparticles for gas-phase photocatalytic hydrogen generation

Anna May-Masnou^1*^, Lluís Soler^2*^, Miquel Torras^1^, Pol Sallés^1^, Jordi Llorca^2^ and Anna Roig^1^

*** Correspondence:** Dr. Anna May-Mansou: amay@icmab.es, Dr. Lluís Soler: lluis.soler.turu@upc.edu

**
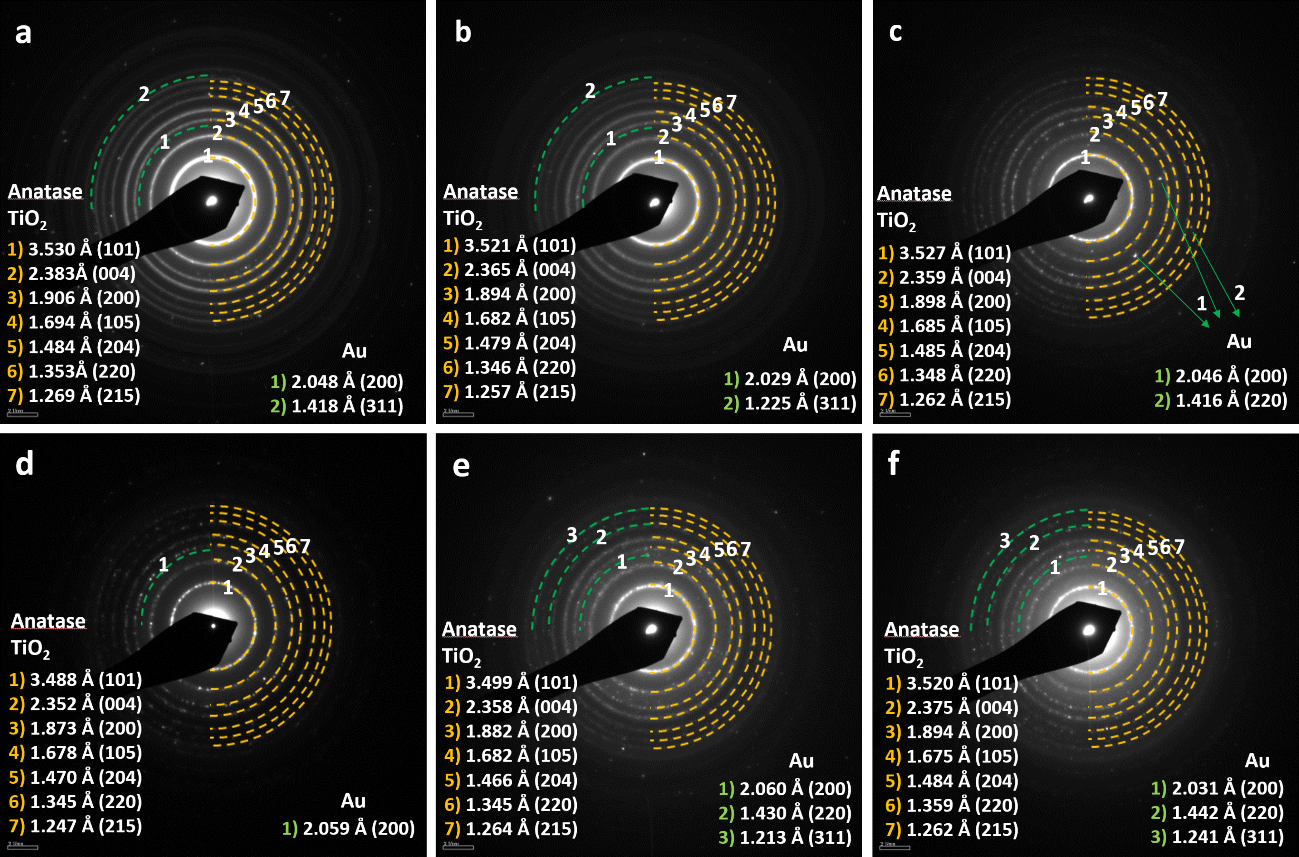
**

**Supplementary Figure 1.** Electron diffraction patterns of sample A **(A)** as-synthesized, **(B)** calcined at 450 °C and **(C)** calcined at 600 °C, and sample B **(D)** as-synthesized, **(E)** calcined at 450 °C and **(F)** calcined at 600 °C.

**Supplementary Table 1.** Size of the Au and TiO_2_ NPs for samples A and B at different calcination temperatures.

|  | **Sample A** | | **Sample B** | |
| --- | --- | --- | --- | --- |
| **Thermal treatment** | **Size (nm)** | **n^[a]^** | **Size (nm)** | **n** |
| **Au** | | | | |
| **Not calcined** | 52 ± 19 | 819 | 8 ± 2 | 651 |
| **400°C** | 57 ± 18 | 152 | N/A^[b]^ | N/A |
| **450°C** | 49 ± 16 | 73 | 13 ± 4 | 157 |
| **550°C** | 51 ± 21 | 30 | 12 ± 3 | 46 |
| **600°C** | 59 ± 21 | 64 | 28 ± 9 | 73 |
| **TiO_2_** | | | | |
| **Not calcined** | 9 ± 2 | 972 | 9 ± 2 | 521 |
| **400°C** | 8 ± 2 | 118 | N/A | N/A |
| **450°C** | 10 ± 2 | 202 | 9 ± 2 | 403 |
| **550°C** | 12 ± 4 | 167 | 10 ± 5 | 194 |
| **600°C** | 13 ± 4 | 59 | 17 ± 5 | 100 |

[a] n = number of NPs counted. [b] N/A = not available

**Supplementary Figure 2.** Size variation with temperature of the thermal treatment for Au and TiO_2_ NPs.


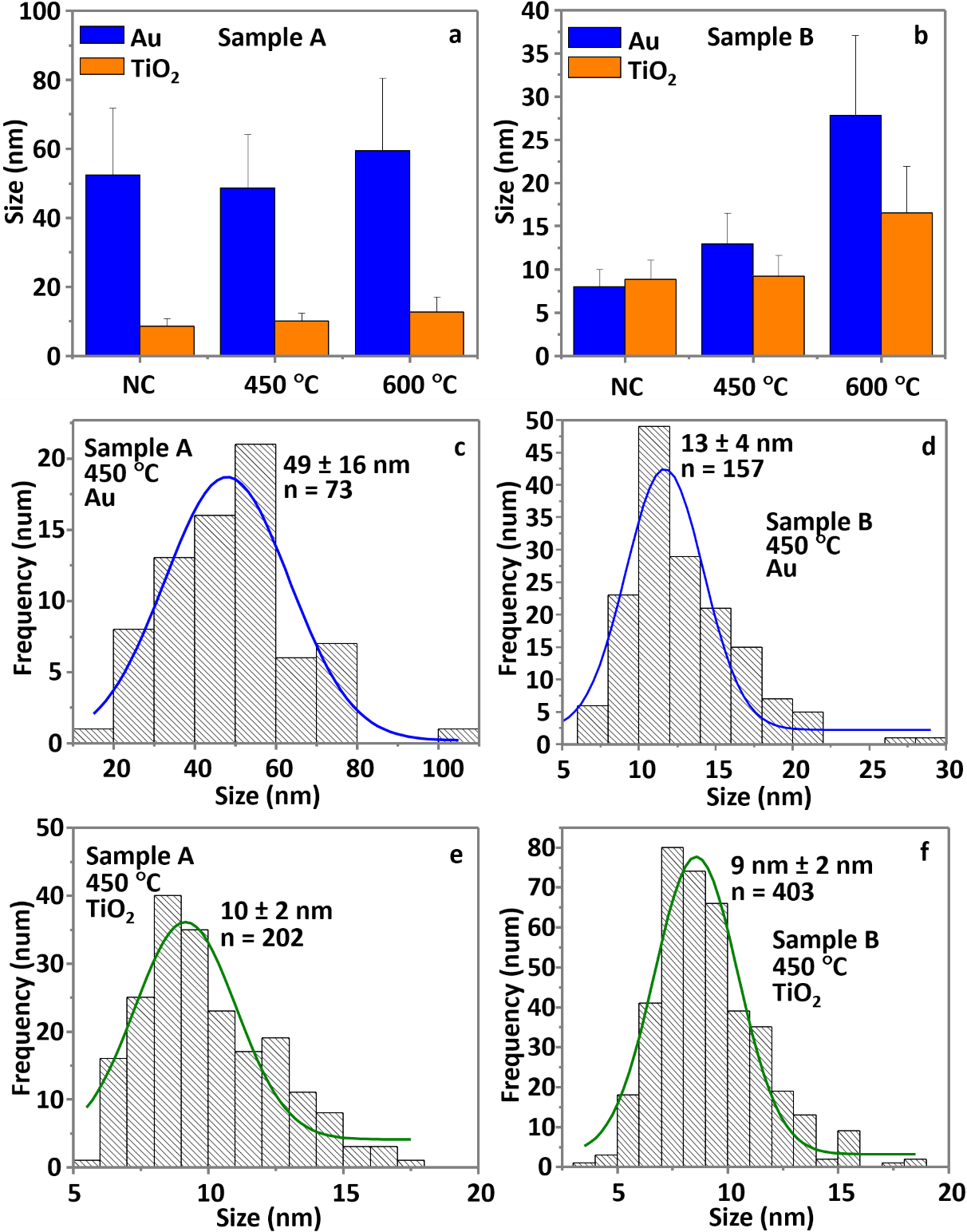


**Supplementary Figure 3.** Size variation with temperature of the thermal treatment for **(A)** Sample A and **(B)** Sample B. Histograms for particle size of samples calcined at 450 °C: **(C)** Au NPs in Sample A, **(D)** Au NPs in Sample B, **(E)** TiO_2_ NP in Sample A, **(F)** TiO_2_ NPs in Sample B.

**Supplementary Figure 4.** TGA of samples A and B as synthesized, and of PVP in air. The mass lost during the analysis of samples A and B corresponds to the PVP, whereas the mass remaining corresponds to Au and TiO_2_ and a fraction of not decomposed PVP. The dry residue for sample A is around 80 wt%, whereas for sample B is 77 wt%. This involves that of the total PVP present in the initial sample (31 wt% in sample A), only 64.5 wt% was decomposed. In sample B, the values are similar, 60.5 wt% of the PVP was decomposed. PVP decomposition started at 350 °C and finished around 470 °C. On the other hand, when pure PVP is treated under the same conditions, only a small residue around 5 wt% remains, so almost 95 wt% of the sample is decomposed, and the starting decomposition temperature is somewhat higher, around 375 °C. The TiO_2_/Au NPs act as catalyst for PVP decomposition, leading to a different decomposition behavior than for pure PVP. This is in agreement with the results obtained when studying the decomposition of Pt-PVP (PVP of 40 k) **(Du et al., 2006**). We considered these values to determine the mass of PVP that remains at each temperature, to express correctly the photocatalytic results as a function of metallic Au or TiO_2_ mass.

**Supplementary Figure 5.** Infrared spectra of **(A)** pure PVP, PVP-covered TiO_2_ NPs, sample A (with PVP), and sample A calcined at 450 °C, and **(B)** pure PVP, PVP-covered TiO_2_ NPs, sample B (with PVP), and sample B calcined at 450 °C. Compared with the spectra of pure PVP, the characteristic resonance peaks in the spectra of PVP-covered TiO_2_ NP, and of samples A and B around 3400 cm^-1^ and 2850 cm^-1^, and 1421 cm^-1^, assigned to ‑OH and ‑CH_2_ stretching, and ‑CH_2_ bending, respectively, present no relevant changes. The stretching vibration peak of C=O, originally at 1658 cm^-1^ in pure PVP, showed a slight red-shift to 1647 cm^‑1^ for TiO_2_ NP, and to 1646 cm^‑1^ for sample A and 1645 cm^-1^ for sample B, revealing the formation of a weak donor-acceptor type interaction by transfer of nonbonding electrons of the O-atom of PVP molecules to the NPs surface. The stretching vibration peaks of C-N, originally at 1072 cm^-1^, were strengthened and shifted to 1087 cm^-1^ in the TiO_2_/Au nanostructures (samples A and B) although the peak at 1285 cm^-1^ did not change. The IR spectra of the calcined samples at 450 °C proved that almost all the PVP was removed, since only a small peak on 1630 cm^-1^, corresponding to the C=O stretch, was present, but there was no presence of any other peaks, except from a small –OH around 3400 cm^‑1^, indicating the hydration of the NPs.


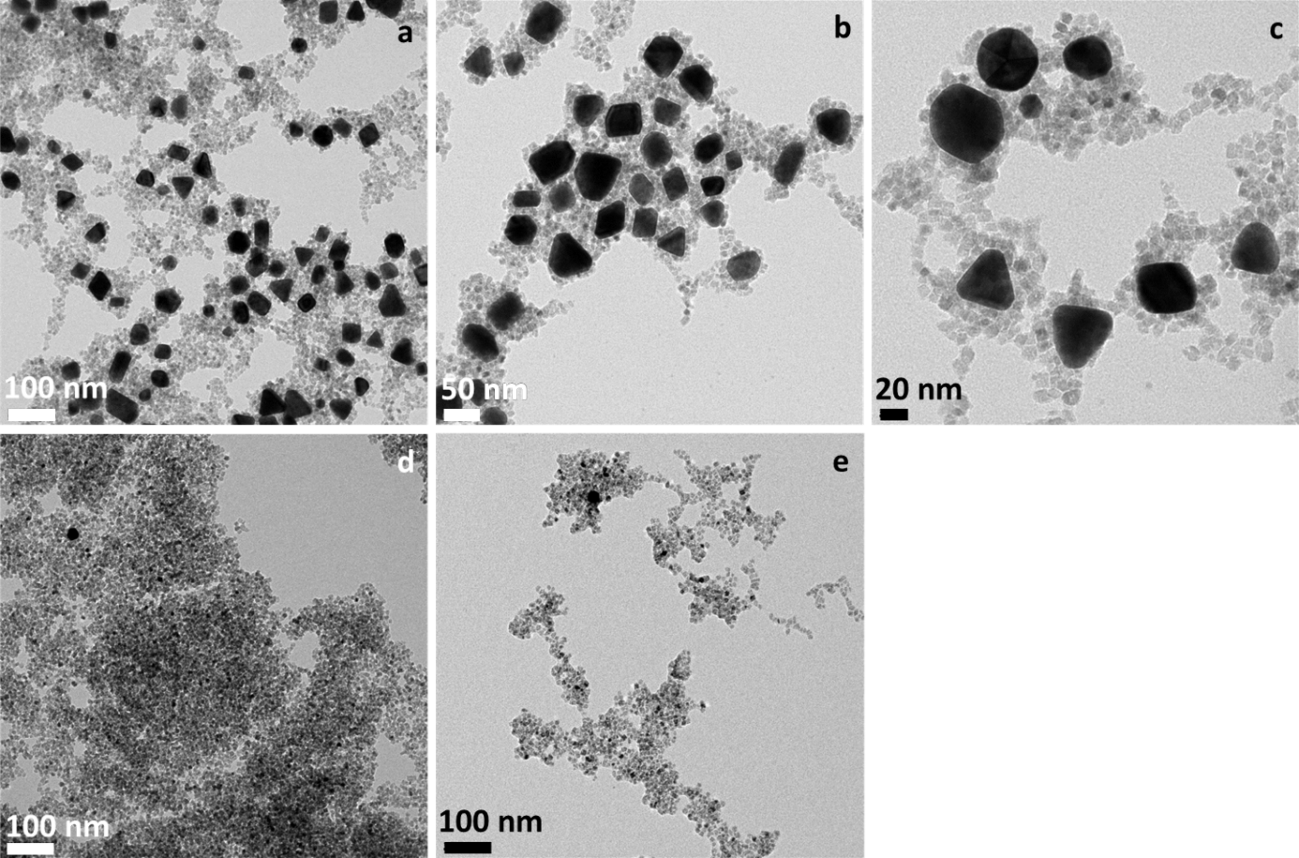


**Supplementary Figure 6. (A,B,C)** TEM images of purified sample A*. **(D)** TEM image of 1st supernatant after centrifuging sample A. **(E)** TEM image of the 4th and last supernatant after centrifuging sample A.

| 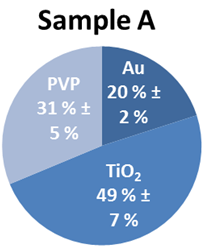 | 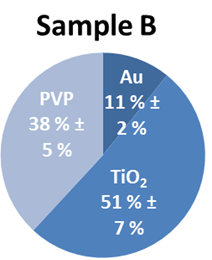 |
| --- | --- |
| 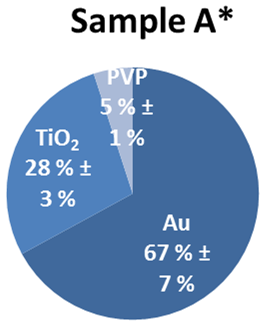 | 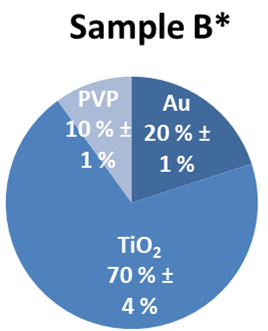 |

**Supplementary Figure 7.** Chemical distribution of samples A and B, and purified A (A*) and B (B*). Percentages are in wt%.

**
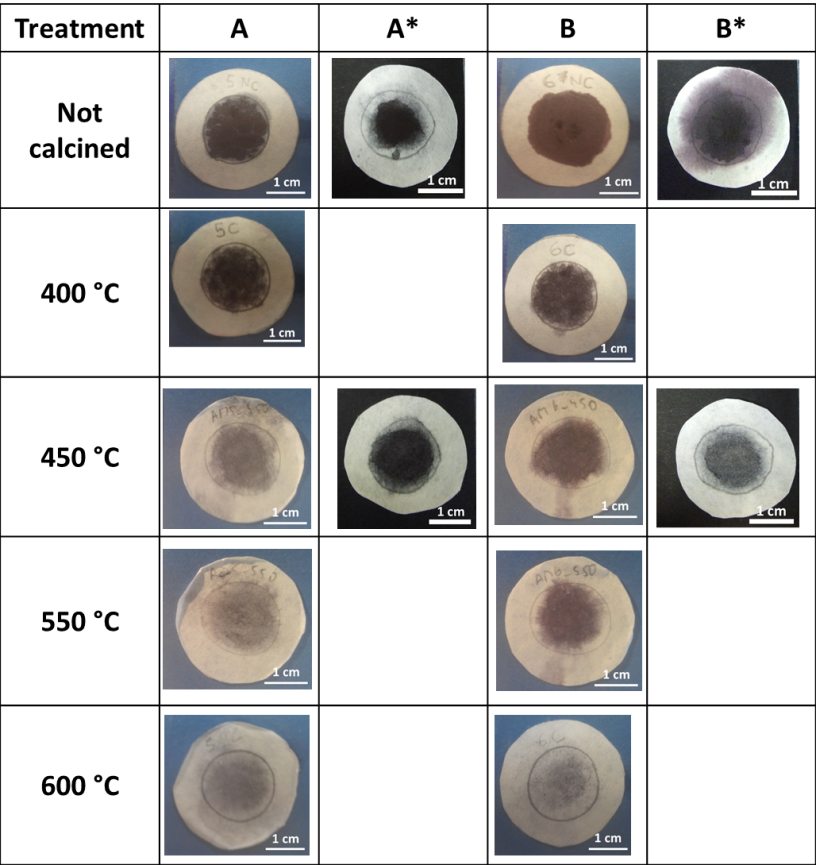
**

**Supplementary Figure 8.** Samples used for the catalytic experiments.

**
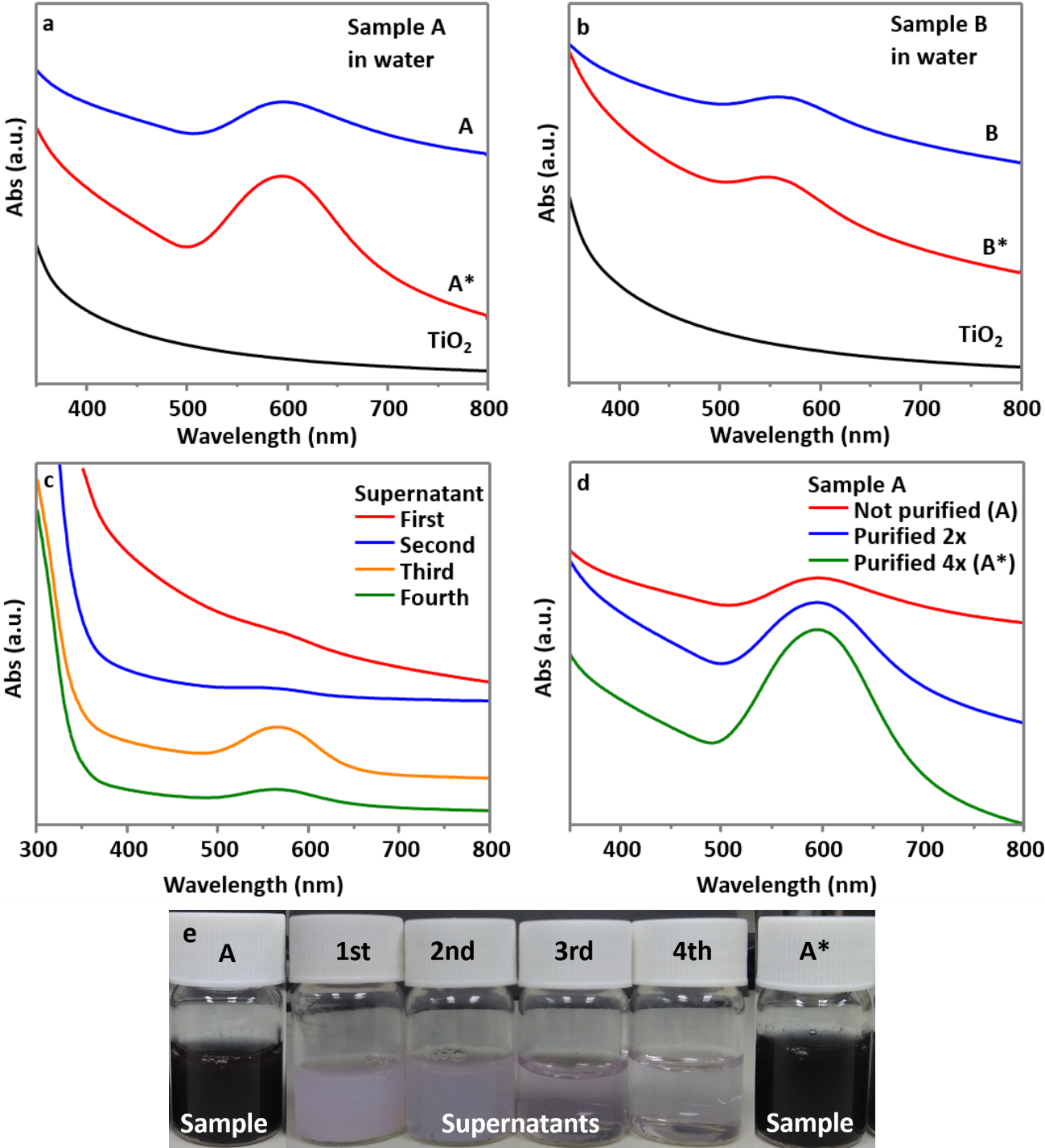
**

**Supplementary Figure 9.** UV-Vis spectra between 350-800 nm wavelength of **(A)** sample A and **(B)** sample B as-synthesized, and purified (A*, B*) and of TiO_2_ before adding the gold precursor, **(C)** of the supernatants obtained from the purification of sample A, **(D)** of sample A as-synthesized (A), purified after 2 centrifugations, and purified after 4 centrifugations (A*). **(E)** Images of sample A as-synthesized, of the 4 supernatants, and of the final purified sample (A*).

**Apparent Quantum Efficiency (AQE):** The AQE was calculated following the equation$AQE=\left( {2n_{H_{2}}}/{n_{p}} \right)\cdot100$, where *n_H2_* is the number of molecules of H_2_ generated and *n_p_* the number of incident photons reaching the catalyst (**Yu et al., 2013**). The number of incident photons can be calculated by $n_{p}={E_{T}}/{E_{p}}$, where *E_T_* is the total energy reaching the catalyst and *E_p_* is the energy of a photon. *E_T_* can be calculated by $E_{T}=PSt$, where *P* (W·m^-2^) is the power density of the incident monochromatic light, *S* (m^2^) is the irradiation area and *t* (s) is the duration of the incident light exposure. *E_p_* can be calculated by $E_{p}={hc}/{}$, where *h* is the Planck’s constant, *c* the speed of light and λ (m) is the wavelength of the incident monochromatic light. The number of hydrogen molecules can be calculated as $n_{H_{2}}=nN_{A}$, where *n* are H_2_ moles evolved during the time of light exposure (*t*), and *N_A_* is the Avogadro constant.

In our experimental conditions, the wavelength of the incident light was λ = 365 nm, the power density of the incident light at the paper surface was P = 81.7 mW·cm^-2^ and the irradiation area was S = 2.54 cm^2^

**References**

Du, Y.K., Yang, P., Mou, Z.G., Hua, N.P., and Jiang, L. (2006). Thermal decomposition behaviors of PVP coated on platinum nanoparticles. *Journal of Applied Polymer Science* 99(1)**,** 23-26. doi: 10.1002/app.21886.

Yu, Y.G., Chen, G., Hao, L.X., Zhou, Y.S., Wang, Y., Pei, J., et al. (2013). Doping La into the depletion layer of the Cd(0.6)Zn(0.4)S photocatalyst for efficient H(2) evolution. *Chem Commun (Camb)* 49(86)**,** 10142-10144. doi: 10.1039/c3cc45568h.
